# Where to Combat Shrub Encroachment in Alpine Timberline Ecosystems: Combining Remotely-Sensed Vegetation Information with Species Habitat Modelling

**DOI:** 10.1371/journal.pone.0164318

**Published:** 2016-10-11

**Authors:** Veronika Braunisch, Patrick Patthey, Raphaël Arlettaz

**Affiliations:** 1 Conservation Biology, Institute of Ecology and Evolution, University of Bern, Baltzerstrasse 6, CH-3012, Bern, Switzerland; 2 Forest Research Institute of Baden-Wuerttemberg FVA, Wonnhaldestrasse 4, D-79100, Freiburg, Germany; 3 Canton de Vaud, Direction générale de l'environnement (DGE), Conservation de la faune, Ch. du Marquisat 1, CH-1025, Saint-Sulpice, Switzerland; 4 Swiss Ornithological Institute, Valais Field Station, Rue du Rhône 11, CH-1950, Sion, Switzerland; Universidade de Aveiro, PORTUGAL

## Abstract

In many cultural landscapes, the abandonment of traditional grazing leads to encroachment of pastures by woody plants, which reduces habitat heterogeneity and impacts biodiversity typical of semi-open habitats. We developed a framework of mutually interacting spatial models to locate areas where shrub encroachment in Alpine treeline ecosystems deteriorates vulnerable species’ habitat, using black grouse *Tetrao tetrix* (L.) in the Swiss Alps as a study model. Combining field observations and remote-sensing information we 1) identified and located the six predominant treeline vegetation types; 2) modelled current black grouse breeding habitat as a function thereof so as to derive optimal habitat profiles; 3) simulated from these profiles the theoretical spatial extension of breeding habitat when assuming optimal vegetation conditions throughout; and used the discrepancy between (2) and (3) to 4) locate major aggregations of homogeneous shrub vegetation in otherwise suitable breeding habitat as priority sites for habitat restoration. All six vegetation types (alpine pasture, coniferous forest, *Alnus viridis* (Chaix), *Rhododendron*-dominated, *Juniperus*-dominated and mixed heathland) were predicted with high accuracy (AUC >0.9). Breeding black grouse preferred a heterogeneous mosaic of vegetation types, with none exceeding 50% cover. While 15% of the timberline belt currently offered suitable breeding habitat, twice that fraction (29%) would potentially be suitable when assuming optimal shrub and ground vegetation conditions throughout the study area. Yet, only 10% of this difference was attributed to habitat deterioration by shrub-encroachment of dense heathland (all types 5.2%) and *Alnus viridis* (4.8%). The presented method provides both a general, large-scale assessment of areas covered by dense shrub vegetation as well as specific target values and priority areas for habitat restoration related to a selected target organism. This facilitates optimizing the spatial allocation of management resources in geographic regions where shrub encroachment represents a major biodiversity conservation issue.

## Introduction

Over centuries the mountain ecosystems of Central Europe have been shaped by extensive agricultural practices such as grazing and meadow harvesting, which have created semi-natural open landscapes characterized by a highly heterogeneous, biodiversity-rich vegetation mosaic [1]. The progressive abandonment of these traditional farming practices is now giving way to widespread forest and shrub vegetation encroachment [2], which threatens biodiversity [3, 4]. The effects are particularly pronounced within the Alpine treeline altitudinal belt, which–due to its highly diverse ecotone structure–harbours a great variety of plant and animal species [5]. Although this process is exacerbated by climate change, land use change still remains the major driver of vegetation ingrowth below the natural treeline, notably contributing to most of the observed woody plant upwards shifts [6]. To counter the erosion of habitat complexity and associated biodiversity in Alpine timberline ecosystems targeted restoration measures are required, which calls for methods that provide not only area-wide information about prevailing vegetation conditions, but also quantitative target values for management interventions, while spatially identifying priority areas for these interventions.

One of the primary focal species of habitat restoration management in Alpine timberline ecosystems is the black grouse. It is considered as a key indicator of structural habitat diversity [7]. Population declines have been recorded throughout its Central European range [8] with habitat loss, habitat degradation [8, 9] and human disturbance [10–13] identified as the main responsible factors. Black grouse reproductive success is primarily determined by habitat quality, notably availability of food and cover [14, 15]. Nesting on the ground, this precocial species is particularly vulnerable during early life stages [14–16]. Breeding habitat must thus offer multiple resources simultaneously: open grassy areas–yielding abundant protein-rich arthropod food for sustaining rapid chick growth–interspersed with woody vegetation that provides shelter under adverse weather and against aerial or terrestrial predators [15]. Encroachment by homogeneous shrub formations, particularly the expansion of green alder (*Alnus viridis)*, has been shown to reduce overall plant species richness and arthropod biomass, which in turn decreases black grouse breeding habitat suitability [17]. Habitat restoration therefore often consists of management interventions in patches of dense shrub formations so as to recreate an heterogeneous mosaic of grassy areas and dwarf shrubs, associated with young and old coniferous trees [7, 18].

Although species’ habitat requirements have been investigated at multiple spatial scales [7, 19] the lack of area-wide spatial information about degrading habitat–notably encroaching shrub–represents an impediment to systematic planning of restoration action. Using the black grouse as a model species, we therefore developed a method that not only identifies patches of dense shrub formations at the landscape scale, but also allows locating where they deteriorate otherwise suitable breeding habitat. We relied on a combination of remote sensing and spatial modelling.

**Fig 1:**
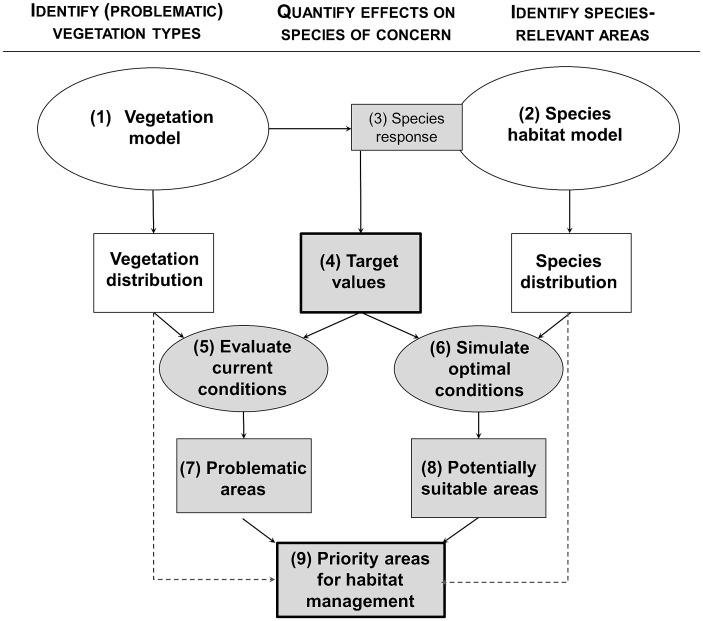
Conceptual framework underlying the approach applied in the present study, showing aims (superscript), methodological steps (ovals) and outputs (rectangles). The modelled vegetation types (1) are used as predictors in species habitat model (2). From the species response curves (3), target values for optimal vegetation configuration are derived (4) which are used to evaluate the current vegetation configuration (5) and simulate potentially suitable habitat when assuming optimal conditions (6). The resulting areas with presently unsuitable conditions (7) within potentially suitable areas (8) are identified as priority areas for management interventions (9). White symbols and dashed arrows illustrate the commonly applied procedure of intersecting the outcome of two independent parallel models; novel interlinking steps are illustrated in grey. Bold-framed symbols represent management-relevant outcomes.

Given the classical trade-off between resolution and extent of digitally available data, spatial predictions across large areas usually come at the expense of precision [20]. Species distribution models therefore frequently rely on proxies, i.e. coarse-grained, but area-wide available data (e.g. climate, topography or human land use), which correlate with crucial habitat features (e.g. vegetation structure or habitat heterogeneity), without necessarily being functionally linked [21]. While this approach can be sufficient for predicting broad-scale species distributions, it fails to deliver appropriate information for on-site habitat management, which requires detailed, fine-grained spatial and quantitative information on existing vegetation features and their deviance from optimal conditions in relation to the management goal. The growing availability of area-wide, high-resolution remote sensing information, derived from satellite or aerial imagery, and airborne laser scanning (ALS), offers the potential to identify vegetation characteristics across large areas with an unprecedented degree of precision [22, 23]. In this study we combine different sources of remotely-sensed information with interlinked spatial models into a framework (Fig 1) that allows to 1) identify and locate key shrub and ground vegetation types at the landscape scale; 2) analyze and predict black grouse breeding habitat selection as a function of these vegetation types; 3) derive species-specific target values for habitat management; 4) locate areas that deviate from optimal vegetation conditions so as to derive priority areas for intervention, notably zones with massive encroachment of dense shrub formations into otherwise suitable black grouse breeding habitat. Our approach is readily transferable to other contexts. It generates high-resolution, quantitative and spatially-explicit information that allows broad-scale systematic planning of habitat management interventions for combatting the detrimental consequences of land abandonment upon biodiversity.

## Methods

### Ethics statement

Species observation data were adopted from existing databases, so no handling or disturbance of endangered species was involved. Vegetation mapping was conducted on public grounds where no permits for were required.

### Study area

**Fig 2:**
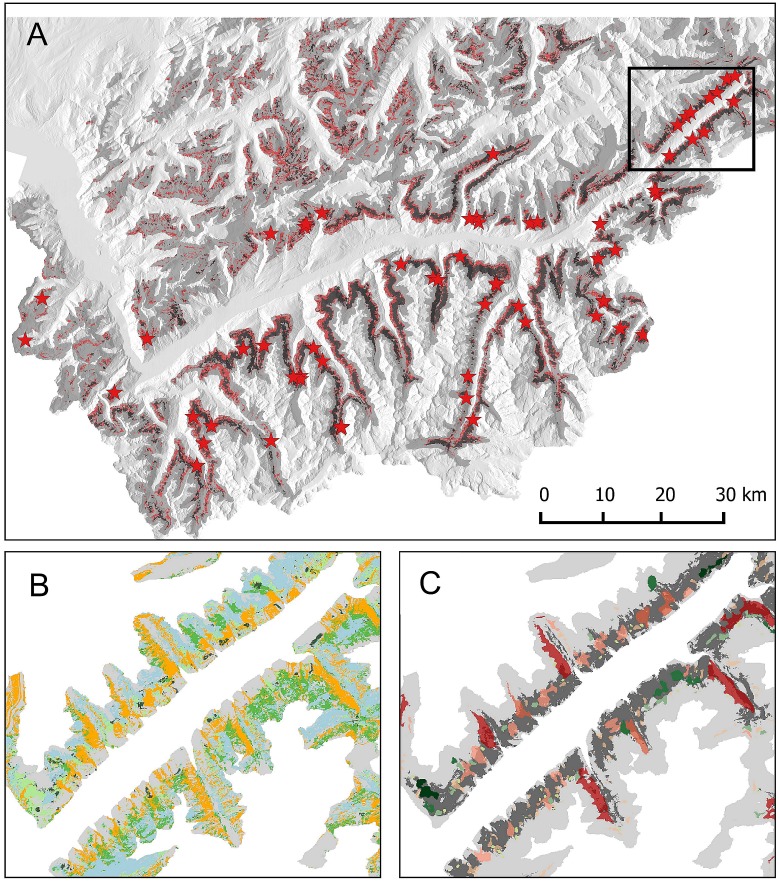
Map of Southwestern Switzerland showing the main outcome of the spatial modelling. A: Distribution of Black grouse breeding locations (red stars), that were used for modelling current (black) and potential (light red) breeding habitat within the timberline belt (1600–2500 m altitude, grey). The black insert in A depicts the region detailed in B and C. B: results of the vegetation classification showing the distribution of alpine pasture (light blue), alder formations (yellow), as well as *Juniperus*-dominated (light green), Rhododendron-dominated (medium green) and mixed (dark green) heathland vegetation. C: Patches of homogeneous heathland (all three types, green) or alder (red) located in potentially suitable black grouse breeding habitat (dark grey). The color intensity of the vegetation patches increases with size, with dark colors indicating larger patches with higher priority for habitat management.

The study was conducted in SW Switzerland (ca 46°10' N, 7°20' E), within an altitudinal belt of 1600–2500 m a.s.l. covering 3117 km^2^ mainly located along the slopes of the Rhône valley and its main tributaries (Fig 2). This altitudinal range encompasses the subalpine treeline, which is dominated by larch *Larix decidua* (Miller), intermixed with Swiss stone pine *Pinus cembra* (L.) and spruce *Picea abies* (L.). The ground layer is dominated by dwarf shrub formations (*Rhododendron ferrugineum* (L.), *Juniperus communis* (L.) and *Calluna vulgaris* (L.)) and grasslands (*Nardus Stricta* (L.), *Calamagrostis villosa* (Chaix)). Alpine pastures in this altitudinal range are mostly grazed by cattle and sheep. The study area includes two topographic and bioclimatic regions: the Pre-Alps and the Central Alps, characterised by subcontinental to continental climate conditions, respectively, with warm and dry summers, and cold, relatively wet winters [24].

### Vegetation model

#### Vegetation data

Vegetation data were collected during field surveys in 2003, 2006 and 2009, focusing on 6 main vegetation types: Alpine pasture (1), three types of heathland (also including Ericacea) dominated by either Rhododendron *Rhododendron ferrugineum* (2) or Juniper *Juniperus communis* (3) or a mix of both (*Rhododendron* and *Juniperus*) (4), green alder (*Alnus vidiris*) formations (5) and coniferous forest (6) (Table 1). Homogeneous patches of these vegetation types that covered at least 625 m^2^ (25x25m) were identified while walking along random transects (N = 28, average length: 2km) distributed along the timberline of the main Rhône valley axis and its lateral tributaries (S1 Fig). Patches were delineated by taking the circumscribing GPS coordinates, and drawn as polygons using ArcGIS 9.3. The resulting polygons were then converted into a raster layer divided in 5x5m cells, which corresponds to the maximum resolution of the predictor variables (see below). In order to avoid pseudoreplication, as some of the predictor variables were only available at a resolution of 25 x 25m, out of a 25x25m block only the most central 5x5m cell (hereafter referred to as “sampling plot”) was retained for further analyses, which resulted in 516 vegetation plots.

**Table 1:** Definition and description of the six vegetation types characterized in the field and used in predictive modelling.

	Habitat type	Code	Classification (Delarze et al. 1999)	Definition
1	Alpine pasture	PASTURE	Nardion strictae, Festucion variae, Poion alinae	> 75% herbaceous plants
2	Rhododendron heathland	RHODO	Rhododendro-Vaccinion	> 75% *Rhododendron ferrugineum*
3	Juniper heatland	JUNI	Juniperion nanae	> 75% *Juniperus communis*
4	Mixed heathland	MIXED	Juniperion nanae + Rhododendro-Vaccinion	> 75% area covered by a mix of *R*. *ferrugineum* and *J*. *communis*, but each species <75%
5	Alder	ALNUS	Alnenion viridis	> 75% of *Alnus viridis*
6	Forest	FOREST	Vaccinio-Piceon, Juniperio-Laricetum, Larici-Pinetum cembrae, Abieti-Piceon	>75% of conferous forest

#### Environmental predictors

Predictors for modelling vegetation types were extracted from satellite images, supplemented with digital topographical information (Table 2). High-resolution information was obtained from SPOT5 satellite images of 2007, which had been corrected for illumination and atmospheric effects using ATCOR3 [25] implemented in PCI Geomatics Geomatica (Version 10.0.3). Radiative transfer in ATCOR3 was calculated using the MODTRAN4 model [26, 27]

**Table 2:** Predictors used for modelling vegetation types, based on satellite imagery (Landsat7 and SPOT5) as well as topography and land-cover characteristics.

Code	Description	Unit	Data source	Resolution
PCA04	PC1 of four colour bands (RGB+NIR) in April 1998	index	Landsat7^1^	25x25m
PCA05	PC1 of four colour bands (RGB+NIR) in May 1998	index	Landsat7	25x25m
PCA08	PC1 of four colour bands (RGB+NIR) in August 1998	index	Landsat7	25x25m
NDVI04	NDVI in April 1998	index	Landsat7	25x25m
NDVI05	NDVI in May 1998	index	Landsat7	25x25m
NDVI08	NDVI in August 1998	index	Landsat7	25x25m
BAND1	1st Reflectance band (red) from August 2007	index	SPOT^2^	5x5m
BAND2	2nd Reflectance band (blue) from August 2007	index	SPOT	5x5m
BAND3	3rd Reflectance band (green) from August 2007	index	SPOT	5x5m
BAND4	4th reflectance band (near infrared) August 2007	index	SPOT	10x10m
NDVI8_5m	NDVI in August 2007	index	SPOT	10x10m
SLOPE	Slope	degree	DEM^3^	25x25m
NORTH	Northness (cosine of aspect)	-1→1	DEM	25x25m
EAST	Eastness (sine of aspect)	-1g1	DEM	25x25m
SOLR	Average annual solar energy per area	Kwh/m^2^	DEM	25x25m
DFOR	Proportion of dense forest	%	Vector25^4^	Vector (~8m)
SHRUB	Proportion of area with bushes	%	Vector25	Vector (~8m)

^1^ ETM+ (Enhanced Thematic Mapper plus) sensor

^2^ HRG (High Geometric Resolution) sensor

^3^DEM: Digital elevation model (SWISSTOPO): http://www.swisstopo.admin.ch/internet/swisstopo/en/home/products/height.html

^4^Vector25: Digital landscape model of Switzerland (SWISSTOPO): http://www.swisstopo.admin.ch/internet/swisstopo/de/home/products/landscape/vector25.html

In addition, to capture general vegetation and land use pattern across the study area we used Landsat images of different seasons summarizing the information embedded in the four bands (red, blue, green, near-infrared) for each of three months representative of the vegetation period (April, May, August) by means of a principal component analysis, retaining only the first component (PC1) for subsequent analyses. Moreover, we calculated the Normalized Differentiated Vegetation Index NDVI [28, 29], based on the relation of spectral information in both the red and near-infrared range. Additional information on slope, exposition (i.e. northness and eastness, defined as cosine and sine of aspect) as well as the annual amount of incoming solar energy were derived from a digital elevation model. Moreover, we extracted the proportion of forest and bushes within a radius of 50m around the focal cell based on the digital landscape map of Switzerland (Vector 25; precision: 8m; Swisstopo 2007).

#### Statistical approach

We used a multinomial logit model to predict the relative probability of presence of each of the six habitat types as a function of the environmental variables, based on all sampling points. First, from among pairs of highly correlated predictors (Spearman’s |rs| > 0.7), we retained the one with the higher explanatory power in univariate models. Subsequently, starting with the full set of retained predictors, the most parsimonious model was identified by applying a stepwise backward selection procedure [30] based on Akaike’s Information Criteria (AIC) [31]. The multinomial logit model assigned to each sampling plot a probability of presence of each of the six habitat types, i.e. six probability values in total. We assessed the predictive accuracy for each habitat type separately based on Cohen’s Kappa maximum (K_max) and the area under the receiver operating characteristics curve (AUC). Moreover, applying the threshold at K_max, dichotomous maps of presence and absence for each of the six habitat types were generated and the sensitivity (i.e. the proportion of correctly predicted presences) was calculated. In a first step, we evaluated the model fit on all sampling plots; secondly, we simulated an independent evaluation by means of three-fold cross validation. Finally, the model was extrapolated to the entire study area (25x25m resolution), excluding “irrelevant” locations, i.e. areas covered by lakes, glaciers, rocks or dense forest as delineated from the Vector25 map.

### Black grouse breeding habitat model

#### Species data

Evidence of breeding black grouse included observations of nests and incubating hens as well as hens leading chicks, recorded between 2000 and 2011. Since breeding events are quite elusive and systematic surveys of reproduction across the whole study area was out of scope, we relied on casual information gathered at the Swiss Ornithological Institute (notably *via* www.ornitho.ch), supplemented by observations compiled by the Cantonal Game Service as well as data from our own long-lasting research programme. Only presence points with a minimum precision of 100m were included, which resulted in N = 67 data points distributed all over the study area (Fig 2).

#### Environmental predictors

As environmental predictors for modelling black grouse breeding habitat we used information on vegetation, climate, topography and human infrastructure (Table 3). All predictor variables were prepared as raster maps (cell size: 25x25m). In order to both capture the environmental conditions prevailing around these observation points and to account for the sampling accuracy of species locations, we calculated means (continuous variables), proportions (Boolean and categorical variables) or densities (for both point and linear features) within a circular moving window with a radius 100m.

**Table 3:** Variables used for predicting black grouse breeding locations. The species’ response to the variables retained in the best model are indicated with (+) for a positive, (-) for a negative and (o) for a unimodal response. The variables’ contributions to the model are provided as average and standard deviation across 5 cross-validation replicates.

Variable code	Description	Unit	Source	Response type	Contribution (%)	Permutation importance
**Topography**						
SLOPE	Slope	degree	DEM^1^	o	7.5 (2.6)	16.6 (7.9)
NORTH	Northness (cosine of aspect)	-1→1	DEM			
EAST	Eastness (sine of aspect)	-1g1	DEM			
ROUGH	Relief roughness (SD of altitude)	m	DEM			
ROCK	Proportion of rocks	%	Vector 25^2^	-	10.0 (1.7)	18.0 (1.4)
**Climate**						
TAVE6	Mean Temperature in June	°C	Worldclim^3^	o	18.7 (0.7)	23.6 (4.0)
PREC6	Mean Precipitation in June	mm	Worldclim	o	0.7 (0.6)	1.0 (0.9)
SOLR6	Mean solar energy per area in June	Kwh/m^2^	DEM			
SOLD6	Mean monthly sunshine duration in June	h	DEM	+	2.7 (0.6)	3.2 (3.2)
**Human infrastructure**						
ROAD	Density of roads	m/ha	Vector 25	-	0.5 (0.2)	0.5 (0.4)
SETTLE	Proportion of settlements	%	Vector 25			
**Vegetation**						
OFOREST	Proportion of open forest	%	Vector 25	+	18.3 (4.6)	3.6 (2.0)
DFOREST	Proportion of dense forest	%	Vector 25	o	14.9 (2.3)	18.5 (5.0)
TREE	Number of single trees	N/ha	Vector 25	+	9.4 (2.6)	7.0 (3.0)
MEADOW	Proportion of alpine meadow	%	Veg. Model	o	0.5 (0.3)	0.4 (0.8)
RHODO	Proportion of Rhododendro-Vaccinion	%	Veg. Model	o	0.7 (0.3)	0.4 (0.5)
JUNI	Proportion of Juniperion nanae	%	Veg. Model	o	1.7 (0.6)	0.3 (0.5)
MIXED	Proportion of mixed Rhododendro and Juniperion heathland	%	Veg. Model	o	10.5 (3.0)	2.2 (1.8)
ALNUS	Proportion of Alnion viridae	%	Veg. Model	o	3.3 (0.3)	4.1 (1.8)
SIDI	Simpson’s diversity index of MEADOW, JUNI, RHODO, MIXED and ALNUS	%	Veg. Model	+	0.6 (0.2)	0.6 (0.6)

^1^DEM: Digital elevation model (SWISSTOPO): http://www.swisstopo.admin.ch/internet/swisstopo/en/home/products/height.html

^2^Vector25: Digital landscape model of Switzerland (SWISSTOPO): http://www.swisstopo.admin.ch/internet/swisstopo/de/home/products/landscape/vector25.html

^3^Worldclim: www.worldclim.org, downscaled.

Information on tree vegetation, i.e. the proportion of open and closed forest as well as the number of isolated trees per hectare, was drawn from the Vector 25 map. Information about above-timberline ground and shrub vegetation (all vegetation types listed in Table 1, except forest) was taken from the vegetation model. In addition, as an indicator of vegetation heterogeneity, we calculated Simpson’s diversity index [32] from the same five vegetation types, again excluding forest, within a 100m radius.

Climate information included the average temperature and precipitation in June, as obtained from the worldclim-dataset [33] (www.worldclim.org), downscaled to a 100m resolution based on the SRTM-V4 digital elevation model (DEM) and the method described in [34]. We also calculated the mean amount of incoming solar energy per m^2^ and the mean sunshine duration in June according to Fu and Rich [35], and based on the DEM. Topography was described by slope, exposition (i.e. northness and eastness, defined as cosine and sine of aspect) as well as the standard deviation of elevation within a 100m radius [36] as an index of terrain roughness. The proportion of rocks served to assess micro-topographic conditions. Information on infrastructure finally comprised the density of roads and the proportion of buildings and settlements.

#### Statistical approach

As only “presence data” were available, we used Maxent, a machine-learning approach based on the principle of maximum entropy [37], adapted for predictive species distribution modelling [38, 39]. The method compares the environmental conditions at the observed species locations with 10’000 locations randomly sampled across the study area. The environmental variables and functions thereof are used as predictors [39]. Each predictor is weighted by a coefficient, which–starting with a uniform probability distribution–is iteratively changed to converge to the probability distribution that maximises the likelihood of the occurrence data, while remaining as close as possible to a uniform distribution (principle of maximum entropy). The algorithm stops after a predetermined maximum number of iterations or when the increase in log likelihood falls below a minimum value. To avoid overfitting, a smoothing algorithm (regularisation) was employed, that constrains the average value for a given predictor to be within the confidence intervals of its empirical average (for detailed information see [38–41]).

Given the number of presence data (N≤80) we used linear, quadratic and hinge features [41], a maximum of 500 iterations and a convergence threshold of 10^-5^. First we ran a model including all predictors with collinearity based on Spearman’s |rs| < 0.7. Subsequently, to reduce and optimise the predictor set, we conducted a leave-one out stepwise jack-knife procedure by systematically excluding one predictor at a time, thereby discarding all predictors that reduced the models’ predictive accuracy [42, 43]. Model accuracy was determined by the area under the receiver operating characteristics curve AUC [44, 45], calculating the mean across 5 cross validation partitions. The final model was converted into a binary map predicting breeding habitat presence and absence, using the threshold that maximized sensitivity plus specificity of the test data, averaged across the cross-validation partitions.

### Priority areas for breeding habitat restoration

In order to identify currently unsuitable breeding habitats that would be suitable if optimal ground and shrub vegetation composition would be given, since all other environmental requirements are met, breeding habitat suitability was recalculated simulating optimal vegetation composition throughout the study area. Therefore we set the ground and shrub layer variables (i.e. RHODO, JUNI, MIXED, ALNUS and PASTURE, as well as the diversity thereof, SIDI, see Table 3) to their average sampling values at the breeding locations.

In a second step, to identify areas unsuitable due to dense shrub encroachment, the frequency of homogeneously vegetated patches of heathland (all three types grouped) and alder, respectively, within a radius of 100m, was calculated. Aggregations of cells containing more than 50% of the same habitat type within this radius, and thus turning unfavourable as drawn from the breeding habitat model’s response curves, were selected and classified according to patch size. Priority areas for management interventions were finally located by intersecting both types of information, i.e. large, homogeneous shrub vegetation patches overlapping with potential breeding habitat. The resulting map thus shows currently unsuitable, encroached patches that would turn suitable if properly managed.

## Results

### Vegetation model

In the 516 plots the most frequently mapped vegetation types were, in decreasing order: Alpine pasture (n = 174 plots, 34%), Alnion viridae (27%), *Juniperus–*(13%) and *Rhodendro*–heathland (12%), coniferous forest (10%). With only 20 sampling plots (4%), mixed heathland was the least abundant (Table 4). The multinomial model showed a good to excellent fit in predicting the six vegetation types, with from 75% (for rhododendron-dominated heathland) up to 100% (for forest) correctly classified presence-plots, Kappa-values ranging between 0.73 and 1.00 and AUC-values always exceeding 0.9 (Table 4). Cross validation generally indicated a good predictive performance on independent data, but was inherently less accurate for vegetation types with small sample sizes, such as mixed heathland (Table 4). Aggregations of dense shrub heathland and alder formations occurred on 94.9 km^2^ and 253.3 km^2^, respectively, corresponding to 3.0 and 8.1% of the study area. Patch size varied greatly within and between vegetation types. The majority of the identified patches of the two above mentioned types were less than 1 ha in size (58% and 56%, respectively). Among the larger (> 1 ha) patches those dominated by aggregations of dense shrub-heathland were on average smaller (median M: 3.3, interquartile range IR: 1.8–6.7) than patches of green alder (M: 4.3, IR: 2.1–11.1). The **v**ariables retained in the multinomial for predicting the vegetation types are presented in S1 Table.

**Table 4:** Performance of the multinomial model for vegetation classification. (A) Number of correctly and erroneously classified vegetation plots belonging to the six habitat types when applying the threshold at maximum Kappa (K_max) for binary classification; (B) model fit indicated by K_max and the area under the receiver operating characteristics curve (AUC); and (C) predictive accuracy over 3 cross-validation replicates.

**(A)**	**PASTURE**	**RHODO**	**JUNI**	**MIXED**	**ALNUS**	**FOREST**
PASTURE	151					
RHODO		48				
JUNI			57			
MIXED				16		
ALNUS					130	
FOREST						53
Erroneous classification	23	16	9	4	9	0
Total	174	64	66	20	139	53
**(B) Model fit**						
Kappa max	0.81 ± 0.03	0.73 ± 0.05	0.85 ± 0.04	0.77 ± 0.07	0.90 ± 0.02	1
Threshold at K_max	0.52	0.475	0.45	0.34	0.465	0.05
AUC	0.97 ± 0.006	0.97 ± 0.007	0.98 ± 0.007	0.99 ± 0.008	0.99 ± 0.004	1
**(C) Predictive accuracy**						
Kappa max	0.69 ± 0.09	0.54 ± 0.02	0.71 ± 0.14	0.47 ± 0.08	0.86 ± 0.02	0.87 ± 0.04
AUC	0.90 ± 0.05	0.91 ± 0.01	0.92 ± 0.08	0.81 ± 0.11	0.96 ± 0.02	0.96 ± 0.02

### Black grouse breeding habitat

**Fig 3:**
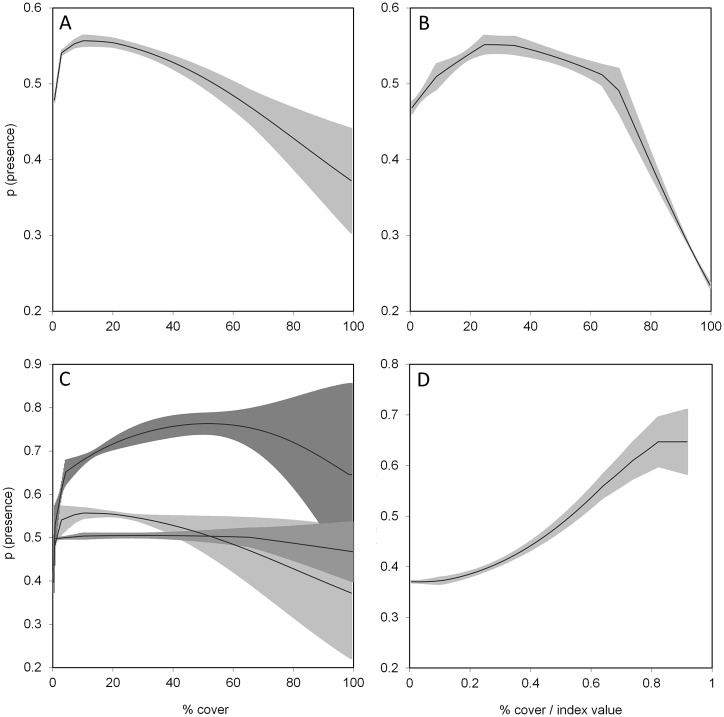
Modelled relative probability (and 95% confidence interval) of breeding black grouse presence as a function of the cover and diversity of five of the six (forest excepted) main vegetation types within a 100 m radius. A: alpine pasture (PASTURE), B green alder (ALNUS), C: *Juniperus*-dominated (light grey), *Rhododendro*-dominated (medium grey) and mixed (dark grey) heathland. D: Simpson’s diversity index drawn from the same vegetation typology.

Black grouse breeding locations were predicted with a high accuracy with a model fit of AUC = 0.925 (SD: 0.007) and an average AUC = 0.885 (SD: 0.042) over 5 cross validation replicates. Breeding habitat suitability was mainly explained by early summer temperature and an intermediate forest cover, i.e. the typical ecotone context of timberline habitats, while ground and shrub vegetation cover contributed only marginally at that spatial scale (Table 3). Black grouse showed a unimodal response towards all shrub vegetation types (i.e. Juniper-, Rhododendron- or mixed heathland as well as Alder formation, Fig 3), with the optimal cover varying between 10% (Juniper-dominated heathland) and 50% (mixed heathland). Moreover, breeding locations were positively associated with a high diversity of vegetation types (SIDI) within a 100m radius.

### Priority zones for habitat management

Under the currently prevailing vegetation conditions 46’947 ha (15%) of the study area provided suitable habitat for black grouse reproduction (Fig 2). In contrast, sites with suitable landscape contexts, irrespective of the prevailing field layer vegetation composition, occurred on a total area of 90’203 ha (29% of the study area). 10% thereof were covered by patches of dense shrub formations, with 5.2% (4672 ha) pertaining to shrub-heathland and 4.8% (4359 ha) to alder formations. These patches, representing the zones where habitat management should be envisioned in priority, corresponded to 49% (of a total of 9490 ha) and 17% (of a total of 25’330 ha) of the area covered by dense shrub-heathland and alder formations, respectively (Fig 2C).

## Discussion

The encroachment of woody plants into progressively abandoned, mostly marginal mountain pastures is a worldwide, large-scale phenomenon [2, 46, 47], often exacerbated by climate change [48]. While resulting patterns of vegetation changes [49] and their impact on biodiversity [4, 17, 50] have already been documented, we were still lacking a cost-effective method that can deliver both spatially-explicit information about areas among the wide, often inaccessible landscape where habitat interventions should be prioritized, and targeted species-specific habitat management recommendations. Our approach fills this gap. It can be easily applied over wide areas, is sufficiently precise to capture main vegetation types and configuration, reveals the fine-grained mosaic requirements of exigent wildlife, and enables delineating areas undergoing habitat deterioration. Therefore we propose a generic conceptual framework for drawing management-relevant information from two parallel, mutually feeding modelling lanes (Fig 1).

### Vegetation model

Remotely-sensed characterization of vegetation has undergone a rapid development thanks to modern satellite and aerial imagery [51]; yet, while there are abundant studies of Alpine ecosystems relating to vegetation productivity [52–54], phenology [55, 56] and structural changes [57, 58], classification of Alpine vegetation types remain scarce. Our model achieved a high accuracy in predicting the six main vegetation associations typically occurring in Swiss Alpine timberline ecosystems, despite the fact that vegetation sampling and satellite data spanned over 6 and 9 years, respectively. Time periods of that duration were chosen so as to be short enough to avoid that vegetation dynamics substantially affects habitat typology at these altitudes [59]; and but long enough to cover the multiple years sampling of black grouse (S2 Fig). Although dense shrub patches may have expanded at their periphery during this time lapse, the core areas of the homogeneously vegetated patches, which we used for model calibration, were unlikely to be altered. Our set of predictors not only consisted of contemporary, high-resolution satellite images (SPOT5), but also of derivate information (PCA, NDVI) drawn from the spectral bands of older, coarse-grained images taken in different seasons, as well as from topography and land-use data. The reason for this choice was that we were not focused on “pure” spectral analysis methodology, but on obtaining wide-scale, cost-effective and accurate vegetation predictions by making best use of the available geo-data. In this respect, auxiliary data, i.e. information that is not directly linked to the recorded vegetation characteristics but bears information about variance in local plant growth conditions and development potential, can enhance the predictive power [60, 61]. This for instance includes topographical features, such as surface roughness, shadowing, slope and aspect which can have strong effects on both plant associations and their reflectance values [62, 63]. We also used the two land cover classes “forest” and “bushes” (extracted from Swiss Vector 25) as predictors, despite the fact that forest was also one of the predicted vegetation types; this relativizes the model’s high discrimination power with regard to forest. Note that—despite not belonging to the vegetation types of interest with regard to our research question—we included “coniferous forest” in the vegetation model to cover the spectral range of all main vegetation associations in the study area thereby achieving a more accurate discrimination of the focal types.

Our method has some drawbacks, though: calibrated using data from homogeneous vegetation patches, the model may be less efficient to localize and delineate mixed vegetation types (e.g. mixed heathland): generally, calibration data that rely on categories (e.g. vegetation types) should be both mutually exclusive and exhaustive, i.e. selected so as to maximize the separability between vegetation types while capturing the full variability within each vegetation type [61]. Given this trade-off, we put more emphasis on separability, since we primarily aimed at detecting homogeneous patches of dwarf woody vegetation. As a consequence, the major patches of homogeneous heathland or alder formations identified by the model may also contain parts of intermixed vegetation. This limitation will have to be compensated by *in situ* adjusted management during the interventions.

### Black grouse breeding habitat model

Shrub removal is a costly operation due to both the difficult if not inaccessible Alpine terrain, and the scarcity of *ad hoc* machinery. Combining spatially-explicit information about massive encroachment zones with information about the habitat requirements and potential distribution of vulnerable wildlife allows confining measures key functional areas. Assessing black grouse breeding over a large spatial extent is a challenging task, however: the species is elusive and the female particularly cryptic. We had thus to rely on casual observations and a presence-only modelling approach. Yet, reliable inference from Maxent requires that presence-only samples stem from random or representative sampling [6]. In our case the fraction of the breeding locations collected with radio tracking were unbiased, whereas casual visual observations might have been biased towards sites with high accessibility or birdwatching hotspots. We consider this a minor issue for three reasons: first, black grouse observations were evenly distributed across the study area, with no spatial aggregation; second, they were negatively associated with the presence of human infrastructure; and third, known black grouse hotspots are likely to reflect suitable breeding areas.

Although we used brood locations with a spatial precision of 100m and characterized environmental conditions within a 100m radius, the species’ response curves we obtained for the field layer vegetation types largely coincided with those obtained from radio tracking at a much finer scale [7]. This independently corroborates the good predictive accuracy of our model, in line with cross-validation and confirms that breeding black grouse hens require a fine-grained, patchy mosaic of different vegetation types, with none of them really dominating the landscape (<50% cover each), not only at the local foraging site scale [7] but also in the wider habitat matrix (this study).

Black grouse response curves provide not only direct target values for habitat management, but can also be used to evaluate the discrepancies between currently prevailing and potential optimal vegetation conditions, with the objective to prioritize sites for management interventions (Fig 1). Although shrub and field-layer formations accounted together for only 17% of model gain (8% permutation importance, Table 3), model extrapolation–under the assumption of suitable field-layer conditions across the entire study area–the amount of suitable breeding habitat doubled. Yet, this difference between current and potential breeding habitat could only partially be attributed to shrub encroachment: only 10% of the potential breeding habitat was in fact covered by large patches of homogeneous shrub vegetation. These 10% represent the zones where management interventions should be prioritized. The remaining fraction may be attributed to an unfavourable constellation of vegetation types, and/or wide non-vegetated, i.e. unsuitable areas (e.g. screes and rocky outcrops).

### Conclusions and management implications

The approach developed here allows a rapid and objective appraisal of zones covered by dense heathland and extended alder formations across wide areas. It furthermore provides both a tool for spatially prioritizing interventions and target values for *in situ* vegetation management measures. This will facilitate the allocation of the scarce resources available for wildlife management. The benefits of confining interventions to areas potentially suitable for the target species become particularly obvious when considering that less than half of the wider, homogeneous heathland patches and only 17% of dense alder formations were indeed located in areas which are potentially suitable for black grouse reproduction.

Despite operated at different spatial scales, all models ([7, 17], this study) so far converge in their target values for improving black grouse breeding habitat. Among heathland dominated matrices, an ideal breeding habitat would consist of dwarf shrub formations (not exceeding 50% cover) and alpine pastures (10–40%), interspersed with single, isolated conifer trees (10 per ha) and small dense groups of rejuvenating conifers (< 3m, 30 per ha). In green alder dominated matrices, the cover of *Alnus* should ideally not exceed 25–30% within a 100m radius (Fig 3; corroborating [17]). Targeted interventions should optimally be implemented over areas of at least 12 ha, which corresponds to the average home range of a chick-rearing hen (13.5 ha; [7]). The selection of encroachment patches for interventions may thus be modulated according to the available financial resources: if budget is too low to efficiently modify the habitat over sufficient areas (at least 12ha) of wide homogenous patches, interventions in smaller patches within a matrix of still fairly good heterogeneity may be more efficient.

As black grouse reproductive success is associated with a high structural and ecological diversity [7, 15], interventions in its Alpine habitat are likely to improve conditions for biodiversity in general. In addition, the vegetation map we produced could also be used to prioritize measures for other species and even other pressing conservation issues (e.g. identifying areas of overgrazing). The method developed here is readily transferrable to any project that aims to combat the negative impact of vegetation change upon rare species.

## Supporting Information

S1 Fig Sampling locations. Plots with homogeneous vegetation (white dots) that were used for modelling the six vegetation types (Table 1) within the timberline belt (dark grey) of the study area.
(PDF)

S2 Fig Timescale of the study. Black grouse breeding occurrences were sampled between 2000–2011 (grey bar). Vegetation sampling (black stars) took place in 2003, 2006 and 2009. High resolution satellite (SPOT5) pictures and Vector25 land-use information were taken from 2007, and amended by coarse-grained information on general landscape patterns obtained from Satellite pics (Landsat7) of April, May and August 1998. The year 1998 was selected as it was the only one for which cloud-free pictures were available for all relevant months.
(PDF)

S1 Table Variables retained in the multinomial model and coefficients for predicting the vegetation types, with ALNUS being the reference category. For vegetation codes see Table 1, variable codes are provided in Table 2.
(PDF)

S2 Table Data used for the vegetation model. Variable codes are provided in Table 2.
(XLSX)
