# Supplementary material for: Where to Combat Shrub Encroachment in Alpine Timberline Ecosystems: Combining Remotely-Sensed Vegetation Information with Species Habitat Modelling
Source: PLoS One. 2016 Oct 11;11(10):e0164318. doi: 10.1371/journal.pone.0164318 (PMC5058552; doi:10.1371/journal.pone.0164318)

S1 Fig. Sampling locations. Plots with homogeneous vegetation (white dots) that were used for modelling the six vegetation types (Table 1) within the timberline belt (dark grey) of the study area.

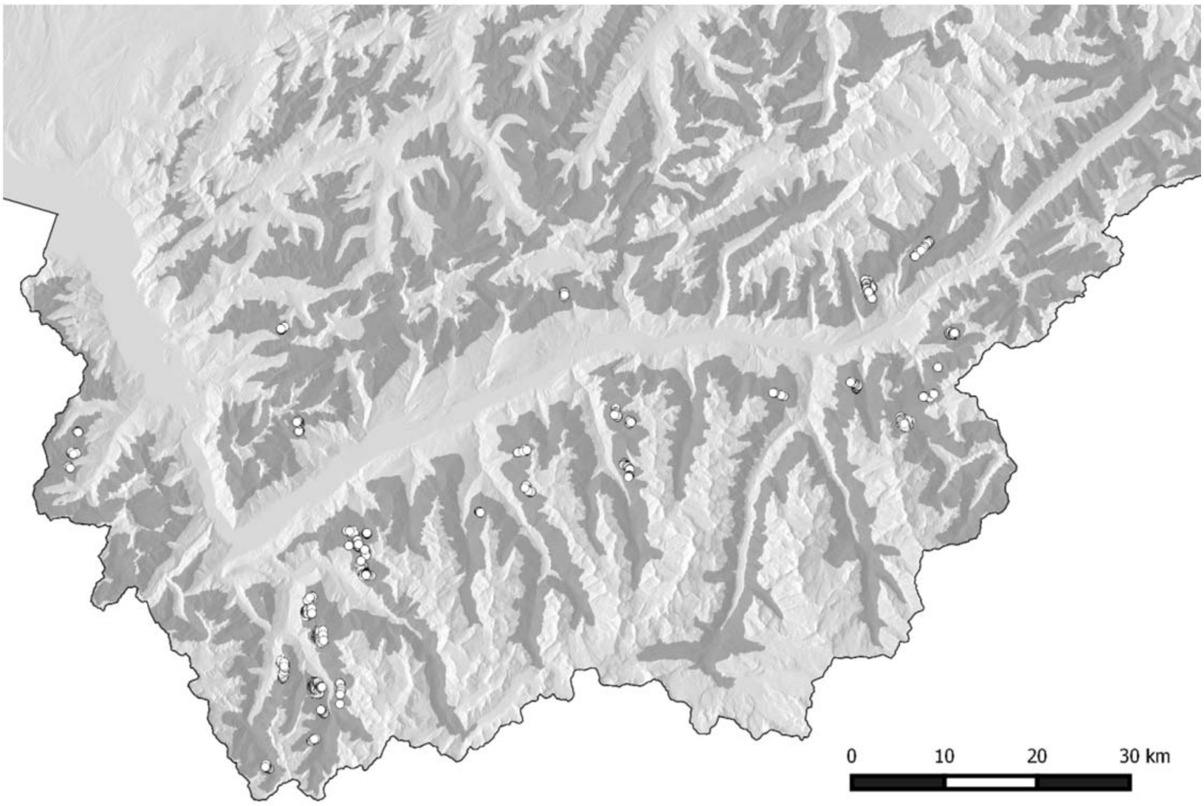

Supplement: S1 Fig — Plots with homogeneous vegetation (white dots) that were used for modelling the six vegetation types (Table 1) within the timberline belt (dark grey) of the study area. (PDF) [file pone.0164318.s001.pdf]
