# Supplementary material for: Where to Combat Shrub Encroachment in Alpine Timberline Ecosystems: Combining Remotely-Sensed Vegetation Information with Species Habitat Modelling
Source: PLoS One. 2016 Oct 11;11(10):e0164318. doi: 10.1371/journal.pone.0164318 (PMC5058552; doi:10.1371/journal.pone.0164318)

S2 Fig. Timescale of the study. Black grouse breeding occurrences were sampled between 2000 – 2011 (grey bar). Vegetation sampling (black stars) took place in 2003, 2006 and 2009. High resolution satellite (SPOT5) pictures and Vector25 land-use information were taken from 2007, and amended by coarse-grained information on general landscape patterns obtained from Satellite pics (Landsat7) of April, May and August 1998. The year 1998 was selected as it was the only one for which cloud-free pictures were available for all relevant months.

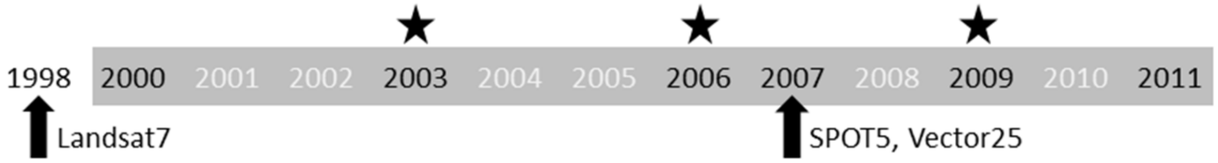

Supplement: S2 Fig — Black grouse breeding occurrences were sampled between 2000–2011 (grey bar). Vegetation sampling (black stars) took place in 2003, 2006 and 2009. High resolution satellite (SPOT5) pictures and Vector25 land-use information were taken from 2007, and amended by coarse-grained information on general landscape patterns obtained from Satellite pics (Landsat7) of April, May and August 1998. The year 1998 was selected as it was the only one for which cloud-free pictures were available for all relevant months. (PDF) [file pone.0164318.s002.pdf]
