# Supplementary material for: Where to Combat Shrub Encroachment in Alpine Timberline Ecosystems: Combining Remotely-Sensed Vegetation Information with Species Habitat Modelling
Source: PLoS One. 2016 Oct 11;11(10):e0164318. doi: 10.1371/journal.pone.0164318 (PMC5058552; doi:10.1371/journal.pone.0164318)
Supplement: S1 Table — For vegetation codes see Table 1, variable codes are provided in Table 2. (PDF) [file pone.0164318.s003.pdf]

S1 Table: Variables retained in the multinomial model and coefficients for predicting the vegetation types, with ALNUS being the reference category. For vegetation codes see Table 1, variable codes are provided in Table 2.

Variable	PASTURE	RHODO	JUNI	MIXED	FOREST
Intercept	-18,427	-16,901	-28,894	-41,828	-90,02
BAND2	0,043	-0,048	-0,053	0,029	-0,227
BAND3^2	0	0	0	-0,002	0,002
BAND4^2	0,006	0,006	-0,01	0,004	-0,181
NDVI05^2	-72,562	22,241	-28,064	-105,239	-254,037
NDVI08	63,119	14,05	91,393	70,894	196,935
NDVI08^2	-73,2	-14,182	-96,92	-88,053	-685,831
NDVI8_5	9,451	26,774	30,393	51,855	-87,381
PCA04^2	0,104	0,009	0,077	0,084	2,004
PCA08^2	-0,118	-0,221	-0,098	-0,339	0,15
EAST	-4,412	-2,918	-5,637	-3,178	-12,371
NORTH	-1,169	2,566	-3,061	2,047	-8,05
SLOPE	0,191	0,536	0,402	1,198	11,272
SLOPE^2	-0,008	-0,012	-0,011	-0,03	-0,164
SOLR	-0,248	-0,156	-0,225	-0,368	-2,718
SOLR^2	0,006	0,001	0,005	0,008	0,042
DFOR	-0,012	0,036	-0,002	-0,367	1,554
DFOR^2	0,001	-0,012	-0,001	0,006	-0,009
SHRUB	-0,239	-3,244	-0,081	-2,343	16,921
SHRUB^2	0,002	-0,929	-0,001	-1,31	-0,952
